# HIV-associated neurocognitive disorder in a KwaZulu-Natal HIV clinic: A prospective study

**DOI:** 10.4102/sajhivmed.v18i1.732

**Published:** 2017-09-26

**Authors:** Jade C. Mogambery, Halima Dawood, Douglas Wilson, Anand Moodley

**Affiliations:** 1 Department of Internal Medicine, Ngwelezana Hospital, University of KwaZulu-Natal, South Africa; 2 Department of Internal Medicine, Infectious Diseases Unit, Grey’s Hospital, University of KwaZulu-Natal, South Africa; 3 Department of Internal Medicine, Edendale Hospital, University of KwaZulu-Natal, South Africa; 4 Department of Neurology, Grey’s Hospital, University of KwaZulu-Natal, South Africa

## Abstract

**Introduction:**

HIV-associated neurocognitive disorder (HAND) is a consequence of HIV infection of the central nervous system. The prevalence ranges between 15% and 60% in different settings.

**Objectives:**

This prospective study determined the prevalence of HAND at a peri-urban HIV clinic in KwaZulu-Natal. Factors associated with HAND were examined, alternate neurocognitive tools were tested against the international HIV dementia scale (IHDS) score and an association between HAND and non-adherence to antiretroviral therapy (ART) was explored.

**Methods:**

Between May 2014 and May 2015, 146 ART-naïve outpatients were assessed for HAND. IHDS score ≤ 10 established a diagnosis of HAND. Functional capacity was assessed using Eastern Cooperative Oncology Group (ECOG) score. Chi-squared test was used to identify risk factors for HAND. The get-up-and-go test (GUGT) and Center for Epidemiological Studies Depression scale – revised (CESD-r) were tested against the IHDS. HIV viral load done six months after initiating ART was used as a surrogate marker for adherence to ART.

**Results:**

The prevalence of HAND was 53%. In total, 99.9% of patients with HAND had no functional impairment. Age > 50 years old was associated with HAND (*p* = 0.003). There was no correlation between the GUGT, CESD-r and the IHDS score. HAND was not associated with non-adherence (*p* = 0.06).

**Conclusions:**

While the prevalence of HAND is high, it is not associated with functional impairment which suggests that asymptomatic neurocognitive impairment is prevalent. Age > 50 years old is a risk factor for HAND. The GUGT and CESD-r are not useful diagnostic tools for HAND. The relationship between HAND and non-adherence should be further explored.

## Introduction

Human immunodeficiency virus (HIV)-associated neurocognitive disorder (HAND) is a consequence of the invasion of HIV into the central nervous system (CNS).^1,2,3,4,5,6^ The proposed pathogenesis involves two mechanisms of neuronal apoptosis.^1,2,3,7^ The first mechanism is by cytokine release from astrocytes in response to infection of the CNS by HIV.^1,2^ The second mechanism involves direct neural damage by HIV proteins.^1,2^ The symptoms of HAND may be subtle or overt and encompass a spectrum of clinical presentations: asymptomatic neurocognitive impairment (ANI), mild neurocognitive disorder (MND) and HIV-associated dementia (HAD).^2,6,8^ Patients with ANI may progress to symptomatic forms of HAND if HIV treatment is not commenced.^9^

The introduction of combination antiretroviral therapy (cART) has led to a decrease in the prevalence of HAND, which ranges between 15% and 60% in different settings.^2,5,10,11,12^ HAD is uncommonly diagnosed in the ART era but milder forms of HAND persist.^3^ A study conducted in Cape Town, South Africa, found a prevalence of 23.5% in a primary healthcare HIV clinic using a battery of detailed neuropsychological, neuropsychiatric and neuromedical tests.^13^ This study also found that HAND was associated with old age and lower education levels. HAND has not been studied in KwaZulu-Natal where the prevalence of HIV approaches 40% according to the South African 2013 national antenatal sentinel HIV prevalence survey.

A range of clinical tests have been used to assess the neurocognitive function of HIV-infected patients.^6,14,15^ These are time-consuming and require expertise from skilled personnel making them impractical for a busy HIV clinic in resource-limited settings.^16^ Furthermore, they often require a patient to have received a formal education which may be a limiting factor in developing countries. The international HIV dementia scale (IHDS), a simple and pragmatic assessment tool developed in the United States of America (USA), showed a sensitivity of 80% and specificity of 55% when tested against standardised neurological and neuropsychological assessments in the USA and Uganda.^17,18^ The validity of the IHDS was tested in a South African healthcare facility and showed a sensitivity of 45% and a specificity of 79%.^19,20^

Kelly et al. studied the association between HAND and adherence to ART in a Malawian adult population. ART adherence was assessed using serum ART drug levels. The study found that HAND was not associated with non-adherence.^21^ An earlier study conducted in Los Angeles found an association between HAND and non-adherence but only in patients more than 50 years old.^22^ The link between HAND and adherence remains a largely controversial subject with conflicting evidence.^23,24,25^

The objectives of this study were to determine the prevalence of HAND, and the functional capacity of those with HAND, in a peri-urban ART clinic in KwaZulu-Natal, South Africa. Factors associated with HAND were studied, and the association between HAND and non-adherence to ART was explored. The utility of two other clinical assessment tools, namely, the get-up-and-go test (GUGT) and Center for Epidemiological Studies Depression Scale – revised (CESD-r) were tested against the IHDS to determine whether they could be used as alternatives to the IHDS in this setting.

## Methods

### Study setting and population

The study was conducted at a regional, peri-urban HIV clinic in KwaZulu-Natal. Consecutive HIV-infected patients who were ART-naïve with a Karnofsky score^26^ greater than or equal to 80 were screened. Those who were older than 65 years old, those with poorly controlled hypertension, a past embolic or haemorrhagic stroke, past CNS opportunistic infections (e.g. meningeal tuberculosis, cryptococcal meningitis and progressive multifocal leukoencephalopathy) and patients with a history of psychiatric illness or current mental healthcare user were excluded.

### Study procedures

All patients who met the requirements and provided written informed consent were enrolled between May 2014 and May 2015.

A trained research assistant administered and obtained the informed consent and performed the neurocognitive tests: IHDS, GUGT and CESD-r. Functional capacity was assessed by the Eastern Cooperative Oncology Group (ECOG) performance status. Baseline CD4 count, co-morbidities and a history of alcohol consumption were captured. The IHDS was performed on each patient. An IHDS score ≤ 10 was used to diagnose HAND. In addition, the GUGT and the CESD-R were performed. All data were captured on a case record form.

Follow-up data including IHDS, GUGT and CESD-r were captured at the 6-month post-ART initiation visit in those patients who were not transferred out of the clinic and those who presented for this follow-up appointment. HIV viral load results were obtained after six months of ART as per South African Department of Health HIV guidelines. A detectable viral load after six months of ART use was used as a surrogate marker for non-adherence. Detectable viral load was defined as an HIV viral load above 20 copies/mL or 50 copies/mL depending on the assay used.

### Statistical analyses

Data were captured and analysed using Microsoft Excel 2010^®^. Descriptive data were summarised as mean, median and proportions. Chi-squared test was used to examine factors potentially associated with HAND and to investigate the association between HAND and a detectable viral load. Correlations between the IHDS and GUGT and the IHDS and CESD-r were assessed using Pearson’s correlation coefficient. Statistical significance was considered at *p* < 0.05.

### Protocol and approvals

The protocol was approved by the University of KwaZulu-Natal Biomedical Research Ethics Committee (ref: BE421/13), KwaZulu-Natal Department of Health and hospital management.

## Results

**FIGURE 1:**
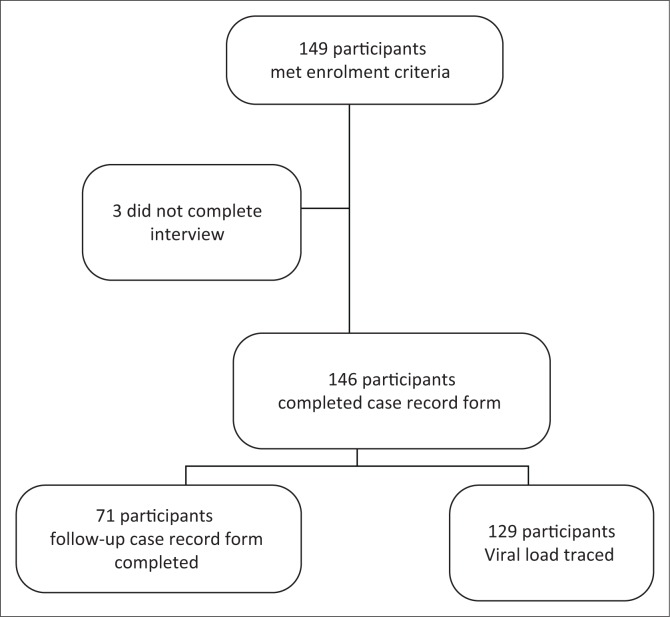
Flow diagram showing enrolment and follow-up.

Between May 2014 and May 2015, 149 patients were enrolled. Three of the patients were unable to continue with the interview because of time constraints; therefore, a total of 146 patients were assessed for HAND. Figure 1 illustrates the enrolment and follow-up of patients.

The baseline characteristics of the 146 patients are displayed in Table 1. Participants were predominantly young women of African descent who were severely immune suppressed.

**TABLE 1:** Baseline characteristics of participants enrolled in the study.

Demographic and clinical characteristics	All participants (*n* = 146)
Sex, % female	54.1
Age, mean (range), years	35 (18–58)
CD4 count , median (IQR), cells/mm³	178 (86–283.5)
Race, % African	99.3
Participants who consumed alcohol, %	42.5

### Prevalence of HIV-associated neurocognitive disorder

**FIGURE 2:**
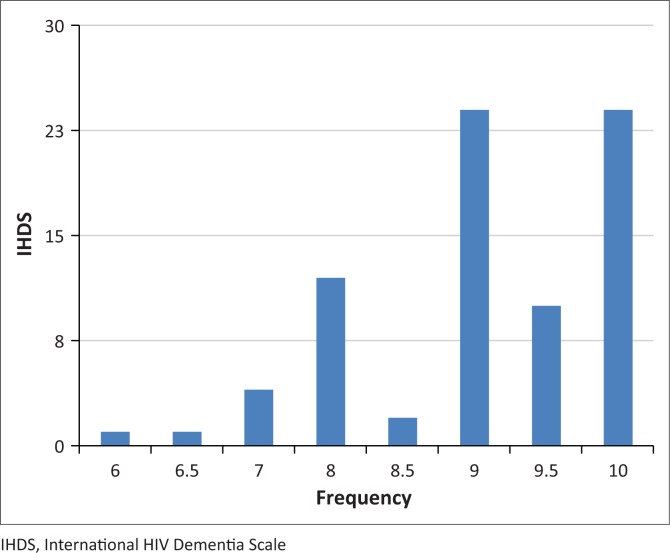
Distribution of International HIV Dementia Scale (IHDS) score among participants with HIV-associated neurocognitive disorder (HAND). The y-axis shows IHDS score and the x-axis shows frequency of the score in the patients who were diagnosed with HAND.

The prevalence of HAND using an IHDS score ≤ 10 was 53% (78/146). The median IHDS score among those with HAND was 9 (IQR 8.5–10). The distribution of IHDS scores among participants with HAND is displayed in Figure 2. Most patients had a score of 9 or 10. The lowest score was 6.

Functional capacity of the patients assessed by the ECOG performance status was 0 in 99.9% of patients with HAND. The GUGT (*r* = -0.12) and CESD-R (*r* = -0.02) did not correlate with the IHDS score.

### Factors associated with HIV-associated neurocognitive disorder at enrolment

The factors that were investigated for their association with HAND are displayed in Table 2. Age greater than 50 years was significantly associated with HAND (*p* = 0.003). There was no association between CD4 count < 200 cells/mm³ and HAND (*p* = 0.14). Remarkably, alcohol use appeared to be protective (*p* = 0.01). Comprehensive data on alcohol use were not collected; therefore, this finding could not be explored further. Nineteen patients had a diagnosis of tuberculosis (TB) at enrolment or follow-up. This was not associated with HAND.

**TABLE 2:** Factors associated with HIV-associated neurocognitive disorder.

Clinical factors	Number in study		HAND		*p*-value
	*n*	%	*n*	%	
Sex	79	54.1	44	56.4	0.5
Age > 50 years old	9	6.2	9	11.5	0.003
CD4 count < 200 cells/mm³	84	57.5	49	62.8	0.14
History of alcohol consumption	62	42.5	26	33.3	0.01
Tuberculosis	19	13.0	13	16.7	0.16

HAND, HIV-associated neurocognitive disorder.

### Impact of HIV-associated neurocognitive disorder on antiretroviral therapy non-adherence

HIV viral load after six months of ART was traced in 129/146 (88%) of patients. A detectable viral load, used as a surrogate marker for non-adherence, was found in 24/69 (35%) with HAND and 12/60 (20%) without HAND. While there appeared to be a trend towards an association between a detectable HIV viral load and the HAND group, the association was not statistically significant (*p* = 0.06). Viral load results were not available for 17 patients who were lost to follow-up.

### Impact of antiretroviral therapy use on neurocognitive test performance

The IHDS, GUGT and CESD-r were re-administered to 71 (48.6%) patients at the 6-month follow-up visit. The improvement on the different test scores after six months of ART use is illustrated in Table 3. In patients with HAND, 70.3% of patients improved their IHDS score by an average of one point. This was a statistically significant improvement (*p* = 0.02). Significant improvements in the other test scores were not found.

**TABLE 3:** Neurocognitive scores after 6 months antiretroviral therapy use.

Neurocognitive test improvement	All patients†		HAND‡		No HAND§		*p*-value
	*n*	%	*n*	%	*n*	%	
IHDS	41	57.7	26	70.3	15	44.1	0.02
GUGT	50	70.4	27	73	23	67.6	0.6
CESD-r	47	66.2	23	62.2	24	70.6	0.4

† , *n* = 71;

‡ , *n* = 37;

§ , *n* = 34.

ART, antiretroviral therapy; HAND, HIV-associated neurocognitive disorder; IHDS, International HIV Dementia Scale; GUGT, get-up-and-go test; CESD-r, Center for Epidemiological Studies Depression scale – revised.

### Impact of antiretroviral therapy use and HIV-associated neurocognitive disorder on CD4 count

The 6-month follow-up CD4 count results of 59 patients were traceable. Of the 59 patients, 52 patients had an improvement in CD4 count with a mean increase of 130 cells/mm³. Although more patients in the HAND group (89% vs. 82%) had an improvement in CD4 count, this was not statistically significant.

## Discussion

This prospective, cross-sectional, descriptive study found a high prevalence of HAND in this peri-urban population. The patients diagnosed with HAND had no functional impairment. Age over 50 years was associated with the presence of HAND. The association between HAND and non-adherence was not significant. There was no correlation between the IHDS and the CESD-r and GUGT scores.

The prevalence of HAND in this community is higher than that previously described in South Africa.^13^ Most patients with HAND were asymptomatic as the functional capacity was preserved in 99.9% of patients. This suggests that ANI in this study population is the predominant manifestation of HAND which may only be diagnosed if patients are screened. ANI increases the risk of symptomatic HAND by two- to six-fold.^9^ It is, thus, important to make the diagnosis as early as possible. Given that the prevalence of ANI is high and that ANI is a risk factor for the development of symptomatic HAND, it is likely that the prevalence of MND and HAD are also high in those with more advanced HIV disease. Unfortunately, this was not assessed in this study.

Age greater than 50 years old was identified as a risk factor for HAND, which is in agreement with previous findings.^27,28^ Exclusion of patients greater than 65 years of age limited the number of elderly patients studied. Inclusion of this age group may have made this variable more significant. The protective effect of alcohol use requires further exploration as insufficient information was collected to explore this further.

CD4 counts at all levels were not significantly associated with the diagnosis of HAND. This finding is not in agreement with other studies.^14,29^ HAD, in particular, has been associated with low CD4 counts. ANI was the predominant form of HAND in this population and may develop irrespective of CD4 count. As none of the patients in this study had evidence of MND or HAD, the association between CD4 count and the symptomatic forms of HAND could not be confirmed.

TB is highly prevalent in this setting; therefore, it is not surprising that a high proportion of patients were HIV and TB co-infected. The association between TB and HAND has not been previously described. In this study population HIV and TB co-infection was not associated with HAND.

Adjunctive tests were carried out to determine whether they were useful to diagnose HAND and could, in fact, replace the IHDS. The GUGT is an easy-to-implement test used to evaluate elderly patients with dementia.^30^ It was postulated that it would be a useful adjunctive test; however, correlation between IHDS and the GUGT was not found. Mood and behavioural changes may be the first symptoms experienced by patients with HAND.^1,2^ The IHDS does not assess these components. We, thus, used the CESD-r to determine levels of depression.^31^ This tool has been used to determine the prevalence of HAND in a Nigerian study and may be implemented by non-medical staff, which makes it an attractive option in resource-limited settings.^32^ Although a significant proportion of patients had a score that suggested a depressed mood, the score did not correlate with the IHDS. Therefore, the GUGT and CESD-r are not recommended for use in the diagnosis of HAND in this setting.

We hypothesised that there would be an association between HAND and non-adherence to ART. Current literature provides conflicting data.^21,22,23,24,25^ A detectable viral load taken six months after ART initiation was used as a surrogate marker for non-adherence to ART.^33^ A statistically significant association between HAND and a detectable viral load was not found in this study.^22,23,24,25^ A larger, more in-depth study may be necessary to explore this association fully. If a conclusive link between HAND and non-adherence is found, it may necessitate a change in ART clinic follow-up and counselling algorithms for patients diagnosed with HAND.

Widespread use of ART has resulted in a significant reduction in the prevalence of HAND.^10^ In this study, there was a statistically significant improvement in IHDS scores in those with HAND after six months of ART use. HAND is not routinely screened for in South African HIV clinics at present; therefore, a diagnosis of ANI may often be missed. However, as the ART programme expands in South Africa, and more patients are commenced on ART earlier in the course of the infection, it is likely that the prevalence of HAND will decrease in keeping with trends in other countries.^10,34,35^

Currently, HAD, the most severe form of HAND, is the only neurocognitive ART eligibility criterion included in the World Health Organization (WHO) clinical staging of HIV and AIDS. South Africa has adopted the ‘ART for all’ guidelines; however, in other developing nations where access to ART is limited, a diagnosis of HAND (symptomatic and asymptomatic) should be an indication for ART initiation. Given the deleterious neurocognitive consequences of untreated ANI, screening is recommended so that this disorder does not go undetected.

The study was limited by selection bias. Patients older than 65 years old and patients with a Karnofsky score of lower than 80 were excluded from the study, which may account for the absence of participants with symptomatic forms of HAND. The WHO clinical HIV stage of each patient was not collected, which may have provided further information on the baseline clinical condition. We were not able to follow-up all the patients who were enrolled because of loss to follow-up and referrals out of the clinic. There were missing data for patients who were lost to follow-up. Additionally, ART drug levels could not be measured to accurately determine patient adherence.

This study provides important information on the prevalence of ANI in this setting where the burden of HIV is high. In addition, it demonstrates that patients with HAND (ANI in particular) may be managed using the same follow-up and counselling algorithms as those without HAND as adherence does not appear to be adversely affected by the neurocognitive impairment.

The inclusion of cerebrospinal fluid HIV viral load to confirm the diagnosis of HAND may be considered for future studies although the use of invasive investigations such as a lumbar puncture for this clinical indication may be a challenge in resource-limited settings. Imaging of the brain was not possible because of cost and access; however, it may be helpful to understand the morphological changes that occur in the brain and should be considered in future studies.

## Conclusion

The prevalence of HAND in this study population is higher than previously reported in South Africa. ANI is the only form of HAND detected in this study population. Age greater than 50 years old is a risk factor for HAND. A CD4 count less than 200 cells/mm³ does not appear to be associated with ANI. This study did not find a relationship between HAND and non-adherence to ART; however, this needs to be explored further.
